# A novel concept for dynamic adjustment of auditory space

**DOI:** 10.1038/s41598-018-26690-0

**Published:** 2018-05-29

**Authors:** A. Lingner, M. Pecka, C. Leibold, B. Grothe

**Affiliations:** 1 Division of Neurobiology, Department Biology II, Ludwig-Maximilians-Universitaet Muenchen, Großhaderner Str. 2-4, D-82152 Martinsried, Planegg Germany; 2 Bernstein Center for Computational Neuroscience Munich, Großhaderner Straße 2-4, D-82152 Martinsried, Germany

## Abstract

Traditionally, the auditory system is thought to serve reliable sound localization. Stimulus-history driven feedback circuits in the early binaural pathway, however, contradict this canonical concept and raise questions about their functional significance. Here we show that stimulus-history dependent changes in absolute space perception are poorly captured by the traditional labeled-line and hemispheric-difference models of auditory space coding. We therefore developed a new decoding model incorporating recent electrophysiological findings in which sound location is initially computed in both brain hemispheres independently and combined to yield a hemispherically balanced code. This model closely captures the observed absolute localization errors caused by stimulus history, and furthermore predicts a selective dilation and compression of perceptional space. These model predictions are confirmed by improvement and degradation of spatial resolution in human listeners. Thus, dynamic perception of auditory space facilitates focal sound source segregation at the expense of absolute sound localization, questioning existing concepts of spatial hearing.

## Introduction

**Figure 1:**
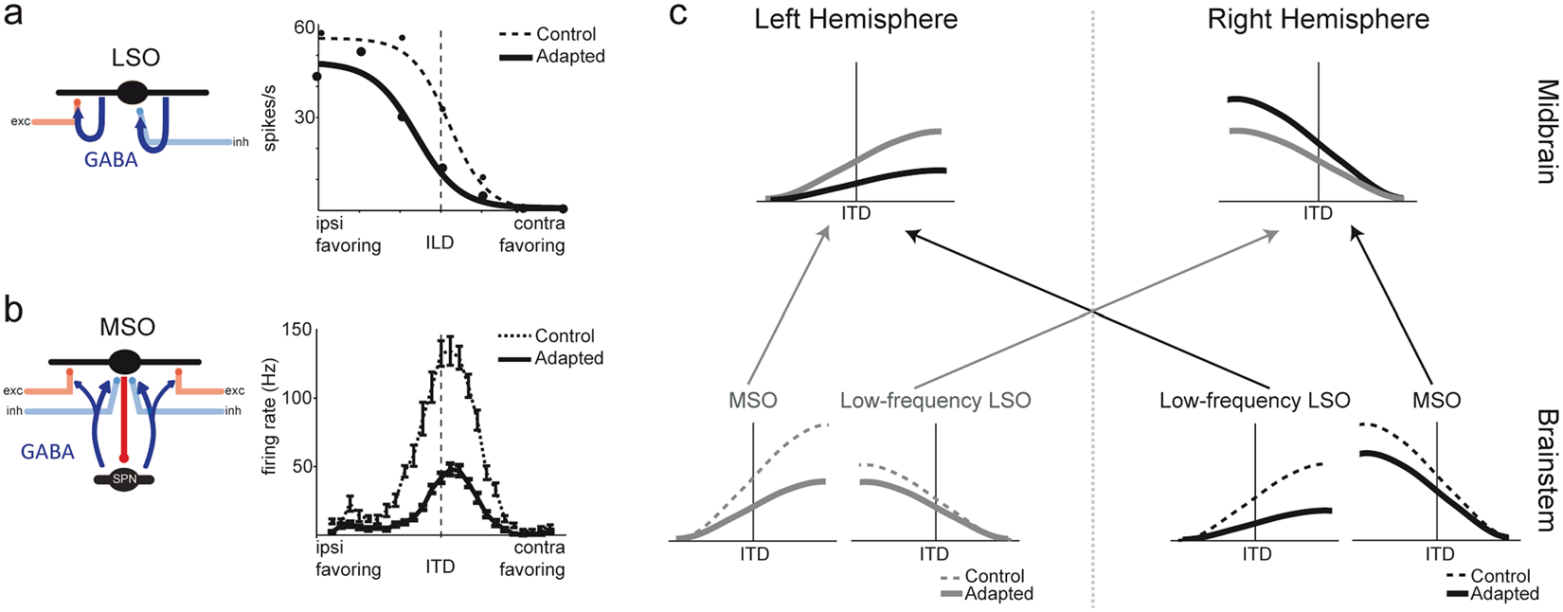
Neuronal coding adapts to stimulus history. (**a**,**b**) Firing rates of both LSO and MSO are modulated by recent stimulus history via a GABAergic feedback loop. Modified from Grothe and Pecka (2014) (**c**) Schematic of the Hemispheric Balanced Model including the effects of hemisperically specific adaptation: sound source azimuth is estimated based on a population vector analysis of MSO and low-frequency LSO for both hemispheres independently. The two estimates are then combined to yield a hemispherically balanced estimate of sound source location at the level of the midbrain.

The localization of sound sources is important for navigation and communication. This ability is neuronally conveyed by an exquisite sensitivity to physical differences of sounds between the two ears (interaural time and intensity differences, ITD and ILD, respectively). For example, human ITD sensitivity permits an angular separation acuity of only a few degrees^1,2^ and is closely matched by the ITD sensitivity found on the level of single neurons in the mammalian auditory brainstem^3–5^. However, how the single cell activity of both ITD and ILD sensitive neurons is transformed into a neuronal code that is underlying the perceptional sensitivity has been debated intensively for decades: Several anatomical and physiological studies in Archosaurs (birds and crocodiles) confirmed the theoretical work of Jeffress^6^, proposing that ITDs are encoded via a so-called labeled-line mechanism resulting in a neuronal place code or map of auditory space^7–10^: Each ITD-sensitive neuron within a population exhibits a narrow receptive field tuned to a specific location in azimuth (=best ITD) with neighboring neurons encoding adjacent positions in space, reminiscent of spatial maps in other sensory modalities. However, in the mammalian ITD detector, the medial superior olive (MSO), as well as in downstream areas, the best ITDs of single neurons do not systematically vary with their location in the nucleus. Rather, MSO neurons exhibited relatively broad ITD sensitivity, where the slopes of single ITD functions span the entire occurring range of ITDs^11–13^. Individual neurons within the MSO of each brainstem hemisphere are similarly tuned (within each frequency band) with opposite tuning characteristics across both hemispheres during control conditions (Fig. 1, dashed lines). A similar hemispheric tuning has also been found for neurons in the Lateral Superior Olive (LSO), the mammalian ILD detector^14^. Owing to this fact, it has been concluded that the ITD and ILD of a sound source is encoded by the relative difference between average activities in the two MSO/LSO populations and this coding strategy is therefore often referred to as two-channel hemispheric (or hemispheric difference) model^11,13,15^.

Common to both theories on sound localization is that they are based on data from single sound source localization in isolation. However, there is psychophysical and phylogenetic evidence suggesting that absolute sound localization may not be the primary task of the mammalian auditory system^16–19^. Correspondingly, it was shown that binaural processing in MSO and LSO is subject to short-term adaptation based on feed-back loops in the mammalian auditory brainstem^18,20,21^, through which the neuronal firing rate is modulated by recent stimulus history (Fig. 1a,b). Specifically, in both nuclei, the inhibitory transmitter GABA (gamma-Aminobutyric acid) is released in activity-dependent manner and binds to pre-synaptic GABA-B receptors to mediated gain adaptation on the time scale of seconds. Such adaptation effects on the single cell level should influence the neuronal coding for acoustic space in the ITD and ILD pathway similarly and thus the perception of space^18,20^.

Indeed, several psychophysical studies demonstrated pronounced context-dependent sound localization in human listeners^17,18,22,23^. That is, the perception of a sound location changes depending on the stimulus context, i.e., the stimulus location and frequency preceding the target stimulus. Specifically, locations of sounds presented in close proximity or at the position of a preceding (tens or hundreds of milliseconds) sound (the “adapter”) are perceptually shifted away from the “real” position. These dynamic effects of stimulus history on auditory space perception were mostly in agreement with the hemispheric difference model of sound localization^18^. Nonetheless, findings of perceptional localization shifts for centralized adapter positions gave rise to the suggestions of an additional midline population channel^24,25^, implying that neither the labeled-line nor the hemispheric difference model might apply to human spatial processing. Thus, short-term history dependency of human ITD perception represents a crucial test for the validity of the assumed underlying processing models. However, so far these models were lacking an account of population responses in the brainstem including adaptation due to stimulus history and hence require refinement (compare Fig. 1a,b)

It is also unclear to what extent compromised absolute sound localization due to history-dependent modulation might serve an ecological purpose. Given the existence of dedicated feedback circuits in the auditory brainstem that are mediating this modulation^18^, we had previously suggested that it might serve to increase spatial sensitivity in complex listening situations. Similarly, Sach and colleagues^26^ and Getzmann^16^ had shown that the presence of a stimulus improves sound source separability for subsequent stimuli at the same spatial position (restricted to midline also in Maier *et al*.^27^). However, changes in absolute localization were not tracked by these studies, rendering a conclusion about the causal role of compromised absolute sound localization for the observed improvement impossible.

Here we studied context-dependent sound localization in human listeners, determined its effect on ITD resolution and used modeling of the data to decipher the underlying spatial code and to predict localization performance in more complex acoustic context. In accordance with previous studies^17,18,22,23^, we found pronounced shifts in the spatial perception of sounds away from the preceding adapter sounds, corresponding to a substantial miss-judgment of absolute sound source positions in the range of tens of degrees of auditory space. For strongly lateralized adapters, theses shifts in perception were restricted to the hemisphere of the adapter location. Such hemispheric confinement of localization errors could not be explained by existing models of auditory space coding (i.e. labeled line or hemispheric difference), even when implementing the afore-mentioned history-dependent neuronal adaptation. Motivated by reports that a unilateral lesion of the midbrain still allows for sound localization in the hemisphere contralateral to the lesion^28–30^, and the fact that the low-frequency limb of the LSO is also ITD sensitive^31,32^, we developed a new hierarchical decoding model in which sound source azimuth is estimated based on population vector analysis for both hemispheres independently. The two estimates are then combined to yield a hemispherically balanced estimate of sound source location (Fig. 1c). This model not only explained the observed absolute localization errors, but also predicted a selective dilation and compression of auditory perceptional space relative to the adapter location. In accordance with this hypothesis, human listeners reported a focal increase in spatial resolution near the adapter on the expense of locations further away. Together, our findings indicate the need for a new concept for the coding of auditory space which is based on the assumption that spatial hearing in mammals serves relative separation rather than absolute sound localization.

## Results

### Selective modulation of spatial perception

**Figure 2:**
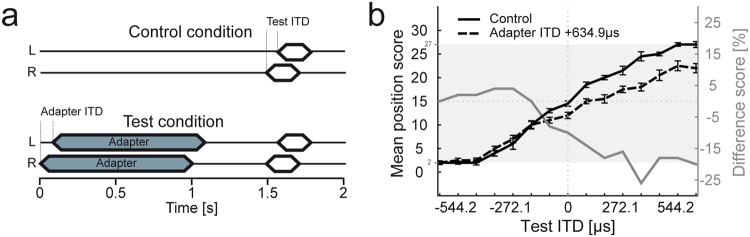
Experimental designs and analyses of perceptional shift. (**a**) Schematic depicting a sample trial sequence to determine the intracranial perception of target tones without and with adapter. Both in the control and test condition target tones (white) were 220 ms in duration and were presented with a randomly chosen test ITD ranging from −634.9 to +634.9 µs in 90.7 µs steps. In the test condition the target tone was preceded by the adapter (1 s in duration) with a pause of 500 ms between adapter and target tone. All stimuli were presented with a frequency of 200 Hz (**b**) Position scores (indication of the listeners’ intracranial target tone perception, with 0 being most left and 30 being most right) without and with adapter (solid and dashed black line) as a function of test ITD for one exemplary subject (data are represented as mean over ten trials for each test ITD, ±S.E.M.). The grey shaded area depicts the individual range (i.e. range between the minimal (2) and maximal (27) mean position score for this subject). To determine the effect of a preceding adapter, the difference score (difference in position scores for each test ITD between control and test condition, normalized to the individual range) was calculated and plotted as a function of the presented test ITDs (grey solid line). Deviations from zero indicate a perceptual shift due to a preceding adapter (negative = shift to the left, positive = shift to the right).

We first determined the localization of target tones with 15 different test ITDs without and with a preceding adapter (Fig. 2a). Listeners (N = 18) had to indicate the perceived intracranial position (stimuli delivered through headphones appear to be within the head) of the target tones by choosing a number from a linear scale between 0 (most left) to 30 (most right). In the control condition, without adapter, mean position scores increased linearly with increasing test ITD (from 2 to 27 for an exemplary subject in Fig. 2b, black line), indicating that listeners were reliably able to report the perceived intracranial position of target tone ITDs, despite of small individual hemispheric asymmetries in the behavioral data (compare mean difference scores for test ITDs of −453.5 and +453.5 µs in Fig. 2b).

In the presence of a preceding, lateralized adapter (ITD of +634.9 µs, 1 s duration, same frequency as the test tone, see Methods), the mean position scores of listeners typically shifted to lower values (Fig. 2b, dashed black line), most prominently within the hemisphere where the adapter was presented. That is, sounds presented within the same hemisphere as the adapter were perceived closer to midline. Thus, in agreement with previous studies that had used similar paradigms^23,33,34^ the perceived intracranial position of target tones was altered by the preceding adapter. This perceptual shift became particularly apparent when the difference score (difference in position scores for each test ITD between control and test condition, normalized to the total range of position scores for each subject) was calculated (grey line, Fig. 2b). Difference scores for test ITDs at and close to the adapter ITD were most negative denoting the strong perceptual shift to the left, i.e. away from the adapter ITD. Difference scores then gradually converged to zero for test ITDs farther away. That is, non-zero difference scores indicate a perceptional shift due to the presence of a preceding adapter.

**Figure 3:**
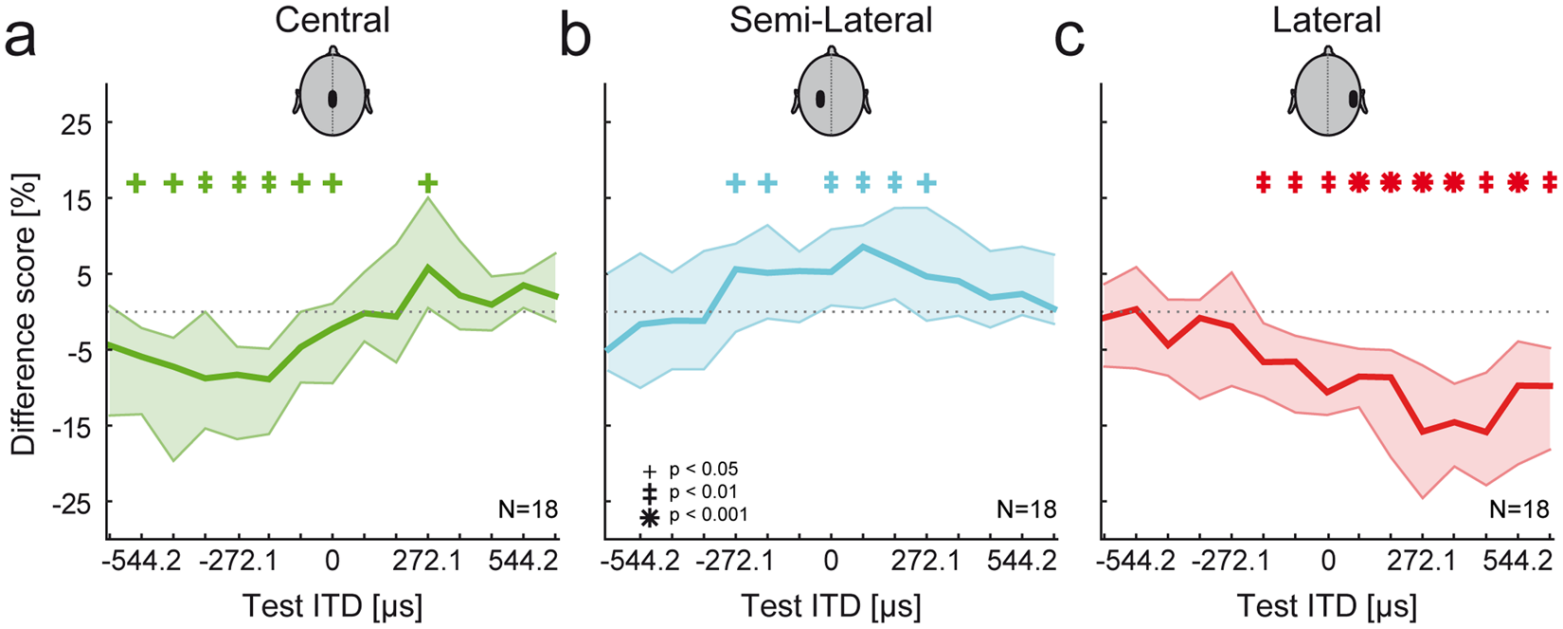
Adapter ITD selectively modulates ITD perception. Median difference scores over 18 subjects (solid line) and interquartile ranges (shaded area) for the three different adapter ITDs as a function of the test ITD are shown. Significant perceptual shifts are indicated by symbols (see legend in panel (**b**), p-values derived from two-sided Wilcoxon signed rank test). (**a**) When a central adapter is presented, the perception of target tones with test ITDs to the left and right of the adapter are affected in different ways: the perception of target tones presented left of the adapter are shifted to the left (negative difference scores), whereas target tones presented right of the adapter are shifted to the right (positive difference scores). (**b**) The presentation of a semi-lateral adapter (ITD of −181.4 µs) leads to a shift to the right (positive difference scores) for target tones with test ITDs ranging from −272.1 to +272.1 µs (except for −90.7 µs). (**c**) The presentation of a preceding adapter with an ITD of +634.9 µs shifts the perception of target tones with test ITDs ranging from −181.4 µs to +634.9 µs to the left (negative difference scores).

To test for ITD-specific effects of the adapter on the perceptual shift, we obtained different scores for three distinct adapter ITDs (central: 0 µs; semi-lateral: −181.4 µs; lateral: 634.9 µs). Generally, all three adapter ITDs lead to a systematic significant shift in the perception of target tone ITDs (Fig. 3, median difference score over all 18 listeners, individual symbols mark perceptual shifts of different significance levels, two-sided Wilcoxon signed rank test). However, the magnitude, extent and direction of the perceptional shift depended on the test condition. When being preceded by a central adapter, the perceived position of target tone ITDs to both sides of the adapter ITD are shifted away from it, leading to opposite directional shifts for both spatial hemispheres (Fig. 3a). Moving the adapter to a semi-lateral ITD (−181.4 µs) also leads to a perceptual shift of target tone ITDs within both spatial hemispheres. In contrast to a central adapter, however, the perceptional shift is now restricted to mostly one direction, i.e. a shift to the right as indicated by a positive difference scores (Fig. 3b). Similar to a semi-lateral adapter the presentation of a lateral adapter (+634.9 µs) leads to a perceptual shift also in only one direction. Beside the effect of adapter ITD on the shift direction, magnitude and extent (number of test ITDs significantly shifted) is also dependent on the adapter ITD being strongest for the lateral adapter, indicating a selectively altered perception of sound location due to the ITD of the adapter.

For all three tested conditions, a preceding adapter moved the perceived stimulus location away from the adapter, indicative of a dynamic process of spatial representation in humans that conflicts with the canonical concept of absolute sound source localization.

### A new model for binaural coding

How can these changes in perception be explained neuronally? Generally, the observed effects of the adapter based on its ITD are in accordance with the recently described negative feedback loop in the auditory brainstem, where a stronger activation of the MSO by more lateralized adapters lead to more pronounced suppression of activity to trailing stimuli^18^. However, the nature of the MSO read-out (“the spatial code”) is still debated^12,35–38^.

**Figure 4:**
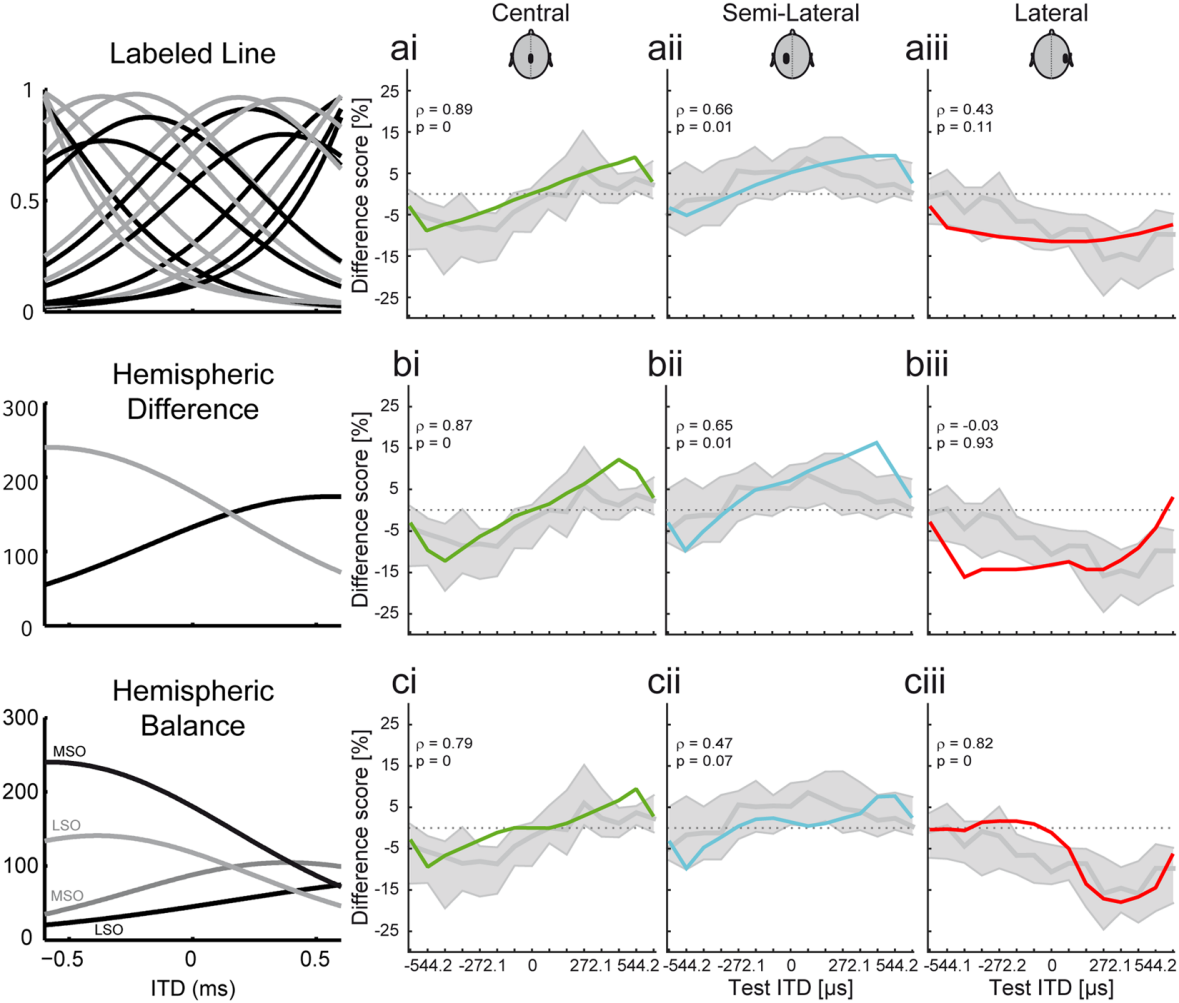
Comparison of neuronal computer modelling and experimental data. (**a**–**c**) Schematics of the three different model concepts (labeled line, hemispheric difference, and hemispheric balance model, respectively). Within each column the corresponding model predictions (solid, coloured lines) and the experimental data (grey) for one of the three different adapter conditions are shown (Pearson’s rank correlation coefficients, ρ, and p-values are given for comparison). (**a**_**i**_–**c**_**i**_) All three models are able to predict the opposing shifts of target tone perception within the left and right spatial hemisphere for a preceding central adapter. (**a**_**ii**_–**c**_**ii**_) Perceptional shifts resulting from a preceding semi-lateral adapter are also reasonable good predicted by all three model concepts. (**a**_**iii**_–**c**_**iii**_) The most striking effect of the three models predictions is found for the lateral adapter: Only the hemispheric balance model restricts the predicted shifts to target tone ITDs within the adapter hemisphere, whereas the two other models erroneously predict a shift over the whole range of target tone ITDs.

Therefore, in order to decipher the underlying coding mechanism we compared our experimental data to the outcome of three different model simulations. Based on anatomical and electrophysiological data of the superior olivary complex, we modeled the population statistics of the MSO and the low-frequency limb of the LSO (lLSO), which is known to also be ITD sensitive^31,32^ (see Methods). The three most important free model parameters (mean best delay, tuning width, and weight function non-linearity) were optimized according to physiological plausibility and overall fitting performance across all models and conditions (see Methods). The simulated neuronal activities including adaptation via negative feedback are the same for all three models. The models only differ in the applied decoding strategy. Two of them are based on classical principles of space encoding, namely labeled-line and hemispheric difference (Fig. 4a,b). For the labeled line model, each simulated neuron votes for its preferred ITD and the estimate is obtained from the respective population vector decoder. For the hemispheric difference model the activities are summed over all neurons in a hemisphere and the resulting population vector decoder is applied to only two dimensions. The third, referred to as “hemispheric balance”, model is based on a novel concept, which performs a population vector analysis in both brain hemispheres independently, leading to two location estimates that are then combined to yield a hemispherically balanced estimate of sound source location. The idea of independent hemispheric estimates is motivated by reports that a unilateral lesion of the midbrain still allows for sound localization in the hemisphere contralateral to the lesion, whereas there are stronger localization deficits in the ipsilateral hemifield^28–30^. Moreover, lesion of the commissure of Probst seems to specifically affect sound localization around midline^39^, where both hemispheres would contribute to a similar extent (according to eq. (1) in Methods). Hemispherically independent processing is further suggested by the stronger ipsilateral connections of the ascending auditory pathway after the level of the olivary complex and particularly the anatomy of the feedback loop in the MSO^18^, which does not cross the hemispheres and thus the resulting adaptive effects only have a unilateral computational purpose.

For all three tested models, the adapter had a significant effect on the lateralization of the target tones (estimated ITD of a test stimulus is based on the population activities in the adapted state derived from eq. (5)). The ability to predict perceptual shifts caused by the presentation of a preceding central adapter was good for all three decoding models (ρ = 0.89 [labeled line], ρ = 0.87 [hemispheric difference], ρ = 0.79 [hemispheric balance]). Similarly, for the semi-lateral adapter, all three models only fit moderately well (ρ = 0.66 [labeled line], ρ = 0.65 [hemispheric difference], ρ = 0.47 [hemispheric balance] Pearson rank correlation, see also Fig. 4a_i_–c_ii_). Crucially, however, the non-uniform perceptual shift as observed after the presentation of a preceding lateral adapter, i.e. a shift that is restricted to target tone ITDs within the adapted hemisphere, was only significantly correlated with the predictions from the hemispheric balance model (ρ = 0.82, p < 0.01 Fig. 4c_iii_). The two other models erroneously predicted strong effects on target tone ITDs within the non-adapted hemisphere (ρ = 0.43, p = 0.11 [labeled line], ρ = −0.03, p = 0.93 [hemispheric difference], Fig. 4a_iii_–b_iii_).

The virtual lack of an effect of the lateral adapter on the contralateral perceptual space is the major feature distinguishing the hemispheric-balance model. The mechanistical underpinnings of this difference is the combination of the two hemispheric estimates by the non-linear weight function g in equation (1), which effectively suppresses the estimate of the less active hemisphere.

**Figure 5:**
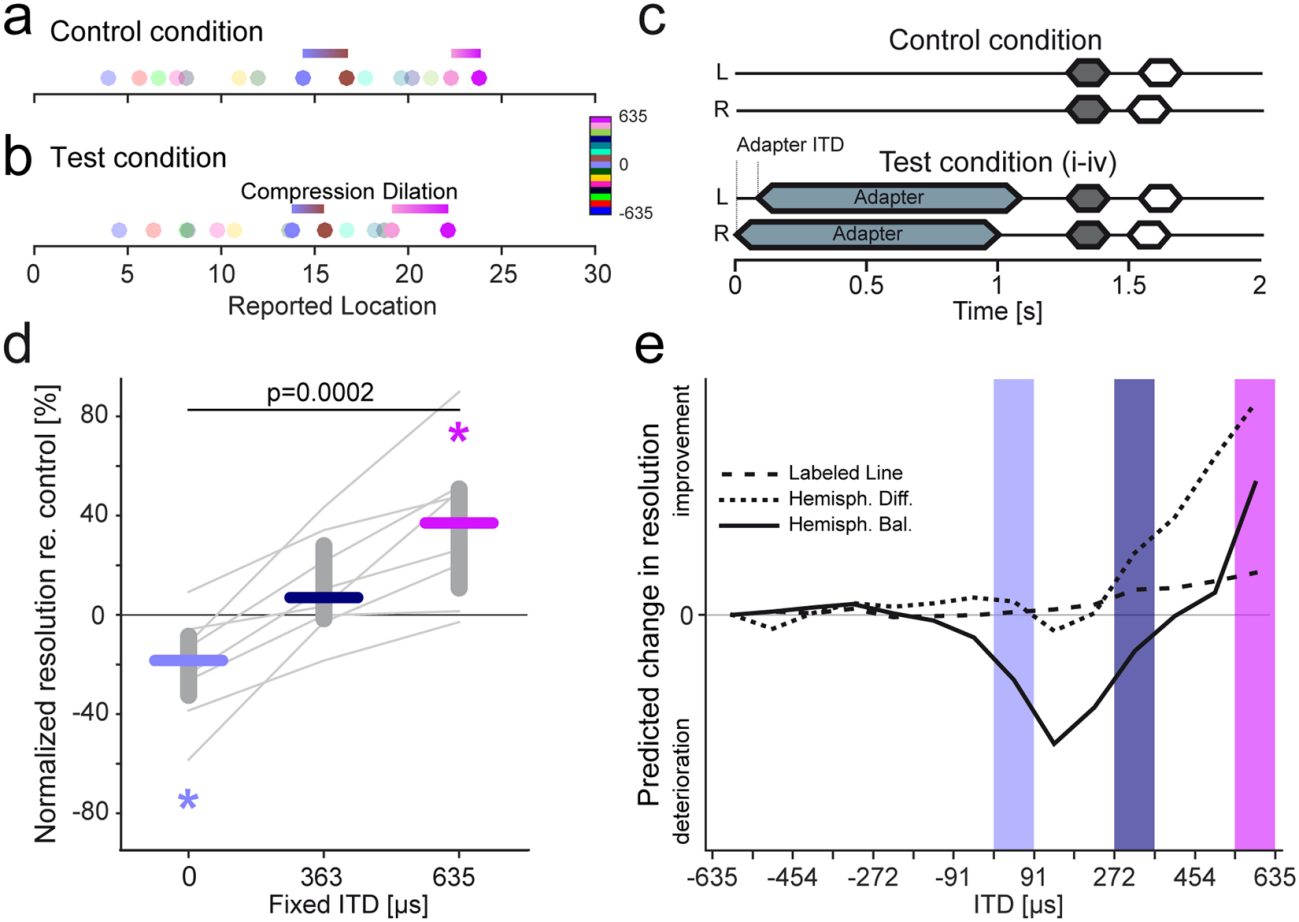
Selective change in resolution in human listeners is captured by the hemispheric balance model only. (**a**) Distribution of reported location values for the 15 test ITDs during the control condition for a representative subject. The resolvability of nearby test ITDs can be approximated by the difference in the corresponding location values (indicated by horizontal bars). (**b**) Location values for the test condition for the same subject show pronounced shifts for test ITDs in the hemisphere ipsilateral to the adapter ITD, yet only marginal shifts around midline. These non-uniform shifts result in a compression, i.e. decreased differences between location values around midline (compare horizontal bar lengths near 0µs ITD in (a) and (b)). Simultaneously, the perception of auditory space close to the adapter ITD is dilated, indicated by increased differences between location values for nearby test ITDs (compare horizontal bar lengths near 634.9µs ITD in (a) and (b)). (**c**) Schematic depicting a trial sequence to investigate the listeners’ ITD resolution without and with adapter (ITD of +634.9 µs). One of the two consecutive target intervals was always presented with a fixed ITD (grey); the other interval was presented with a random ITD (white). The interval containing the fixed ITD was chosen randomly. (**d**) Normalized resolution (re. control) across 8 subjects (colored bars, data are represented as median; grey lines display individual subjects) is plotted as a function of ITD positions. For each subject, ITD resolution at each position was normalized to the resolution at −635 µs for the control and test condition independently. The change relative to the control condition was calculated subsequently. Positive values indicate improved resolution, whereas negative values indicate deteriorated resolution due to the preceding adapter. Overall, the listeners’ resolution increased significantly for locations closer to the adapter (p = 0.00019, Friedman test). Asterisks represents significantly improved or deteriorated normalized resolution for ITD positions close to the adapter position and at 0 µs, respectively (p < 0.05, two-sided Wilcoxon signed rank test). (**e**) The combined compression (deterioration) and dilation (improvement) in spatial resolution at 0 µs and close to the adapter location, respectively, was only captured correctly by the hemispheric balance model. Both other models did not predict the deterioration around midline, due to the missing hemispheric specificity of their fits to the different scores for the lateral adapter (see Fig. 4).

Thus, all three models are able to predict the perceptual changes for a central and semi-lateral adapter reasonably well. However, only the hemispheric balance model was able to correctly reproduce the hemispherical constraint of changes in the listeners’ perception found for the lateral adapter ITD. To further examine the validity of the models in more detail, we next set out to test their predictive power for human auditory spatial resolution. Specifically, we took advantage of these model-specific differences for the lateralized adapter condition, which result in distinct predictions how a listeners’ ability to resolve nearby ITDs should be influenced:

To illustrate the dependency of shifted spatial perception on a listeners azimuthal resolution, Fig. 5a depicts the distribution of reported locations for the 15 test ITDs of the localization task in the control condition for one representative subject. The resolution of nearby test ITDs can be approximated by the difference in the corresponding estimated locations (illustrated by the horizontal bars for two example ITD pairs). This difference corresponds to the relative distance in perceived spatial location. As we have seen (Fig. 3c), the presence of a lateralized adapter resulted in altered location estimates for a subset of test ITDs, namely those in the same hemisphere as the adapter. This asymmetry of the shifts in the listeners’ perception consequently also altered the relative spacing in perceived spatial locations (Fig. 5b) and thus might affect their ability to resolve nearby locations. Specifically, for ITDs near the adapter, the spacing in perceived locations increased, suggesting the possibility of a relatively improved spatial resolution. In contrast, the spacing between nearby location estimates around midline decreased markedly (Fig. 5b), suggesting that the listeners resolution might be impaired at these ITDs. We directly tested this hypothesis by evaluating the spatial resolution in human listeners (N = 8) at three different ITDs.

### The impact of adaptation on sound source separability

To this end, subjects had to report the more lateralized out of two successive target tones in an adaptive tracking paradigm to determine just noticeable differences in ITD (ITD-JNDs) with and without a preceding lateral adapter (+634.9 µs ITD, 1 s duration, same frequency as the test tone, see Methods, Fig. 5c). On average, normalized JNDs (see Methods) during the presence of the lateral adapter were worst compared to control conditions at ITDs around midline and significantly (p = 0.0002, Friedman test) increased with best ITD-JNDs measured close to the position of the adapter. This gradual improvement of spatial resolution is the combined result of a significant improvement of ITD-JNDs close to the adapter and, importantly, a significant (p = 0.02 two-sided Wilcoxon signed rank test) deterioration of ITD-JNDs around midline, as we had hypothesized based on the single subject data (Fig. 5a,b).

To validate the suitability of the various models (and thus the underlying processing strategy), we next used the estimations from the three models to predict changes in the listeners spatial resolution and compared the predicted change in resolution for all three model version to the human psychophysical data at the lateralized adapter condition at the same three test-ITDs (Fig. 5d,e). While all three models correctly predicted an improvement in ITD-JNDs at ITDs close to the adapter, only the hemispheric balance model correctly predicted a deterioration in ITD JNDs around zero µs.

Thus, only the hemispheric balance model predicted the combined improvement and deterioration in ITD resolution as a function of the relative distance tot the adapter (Fig. 5d). Importantly, these location-specific effects are not predicted by the two other models, due to the lack of hemispheric specificity of their different scores for lateral adapters (cf. Fig. 4a_iii_,b_iii_).

Together, these data corroborate the validity of the hemispheric balance model to describe the neuronal processing strategy of mammalian coding of auditory space.

## Discussion

The present study investigated the interrelation between adaptive perception of absolute sound location, sound segregation and the underlying neuronal representation. We found a substantial shift in the subjective perception of sound location away from the preceding adapter, leading to significant errors in the absolute lateralization judgment. This altered perception of sound location resulted in an improved ability to spatially segregate sound sources close to the ITD of a preceding adapter, and in deterioration for ITDs further away. These findings indicate selective compression and dilation of perceptual auditory space coherent with our novel model of spatial coding in mammals based on a population vector analysis performed in both brain hemispheres independently. Together, the results indicate that spatial processing in mammals evolved for relative segregation rather than absolute sound localization.

Similar to other studies examining context-dependent spatial sensitivity (e.g.^18,22,33,40^), we found that test ITDs in close proximity to a preceding adapter are perceptually shifted away from this ITD. The affected hemisphere and also magnitude of perceptional shift was highly dependent on the adapter ITD. Specifically, a highly lateralized adapter (ITD ± 634.9 µs) results in a perceptional shift of all target tones within the ipsilateral hemisphere away from the adapter. Only small or no shifts can be observed for sounds in the hemisphere contralateral to the adapter. For less lateral adapters (ITD ± 181.4 µs) the magnitude of perceptional shift was smaller, however, within both the ispi- and contralateral hemisphere. Thus, the extent of the perceptual shift scaled with the laterality of the adapter. This is also in agreement both with previous findings^22,33^ and with the suggested underlying mechanism in the auditory brainstem, where a stronger activation of the MSO population by more lateralized adapters lead to more pronounced adaptive effects^18^. It is unlikely that the observed resolution improvement/deterioration are effects of altered spatial attention, because we controlled for this through normalization (see Methods) and it has been shown that these effects and the underlying shifts in spatial perception are highly specific for a match between adapter and test tones^18,33^. For the purpose of this study, the time interval between offset of the adapter and onset of the target tone was kept at 500 ms, as this interval has been used before to describe spatial adaptation effects^18^. This rather long-lasting history-dependence of the adapter on test ITDs corresponds well with the likely underlying physiological mechanism, namely a comparatively slow GABA-B mediated modulation of synaptic properties in the MSO and LSO^18,20^. However, at very short time intervals between adapter and target tone (<100 ms), the direction of perceived location shifts can be reversed, possibly due to the additional influence of mechanisms relating to the precedence effect^41,42^.

The present study tested ITD processing of low frequency sounds, representing the most relevant cue and frequency range for human spatial hearing. Stimulus history dependent spatial perception is, however, not only restricted to the low-frequency domain. Perceptional shifts away from the adapter ITD were also found for high-frequency stimuli based on envelope ITDs^43^, as well as based on ILDs^17^. This is consistent with the finding of GABA-B mediated adaptation in the LSO, which is functionally equivalent to the GABA-B mediated adaptation in the MSO. Hence, binaural adaptation of the computation of ILDs and ITDs at the earliest level of binaural processing seem to be a general feature in the mammalian auditory system. The high congruence we found between human perception and models based on physiological parameters determined in rodents^4,5,12,18^ provides further corroboration for an common processing origin of mammalian spatial hearing^19^.

The description of the neuronal code for sound localization, and for ITDs in particular, was subject of several studies (see^13^ for review). The two most prominent models for sound localization (labeled line and hemispheric difference model), however, are not able to fully account for the stimulus driven adaptation found in the present study (Fig. 4a_i–iii_,b_i–iii_). Both models fail to capture the effect of a highly lateralized adapter, because they predict a perceptual shift that affects both hemispheres in contrast to the experimental data showing only a limited effect on the contralateral hemisphere. There have already been studies that refined existing models of auditory space coding in order to predict human psychophysical adaptation data^22,24,25,44^. Kashino and Nishida^22^ implemented a feedback gain control mechanisms in the context of a labeled line ITD processor to explain adaptational effects of sound localization. Other studies^24,25,44^ extended the hemispheric difference model by a third midline channel to account for observed perceptional effects of a central adapter. However, – to our understanding – both the additional feedback gain control mechanism and also an additional channel alone cannot account for the afore mentioned hemispheric specificity, as it would not be affected by lateralized adapters. In contrast, our newly developed hemispheric balance model was designed to capture this hemispheric specificity and also explains the effects of central adapters. Importantly, this model also made specific predictions about selective changes for sound source segregation due to compressive and dilative effects in perceived auditory space. While it has been suggested before that spatially congruent preceding stimuli might improve spatial resolution (^16,26^but see also^27^), no detrimental effects of an adapter on separability were known so far. Crucially, human perception closely followed both model predictions, showing both improvement and deterioration of ITD resolution at specific locations and thus corroborating the hemispheric balance concept as a potential basis for auditory space coding in mammals.

It follows that in contrast to Archosaurs, as shown for birds and crocodilians^7,10,45,46^, that exhibit a hard-wired labeled line coding of auditory space, mammalian auditory spatial processing is dynamic and does not provide an absolute and stable representation of the environment, but emphasizes focal separability of particular sound sources to the detriment of the overall localization ability. These differences might be based on the independent evolution of the middle-ear in birds and mammals and their different starting point in the evolution of spatial hearing about 200 million years ago (for review^19^): While the avian system is inherited from large predatory animals, the ancestors of tympanic ears in mammals are tiny nocturnal prey animals. Potentially, and in contrast to avian predator evolution^47,48^, the dominant evolutionary driving force of the early mammalian localization system was not the faithful mapping of the surrounding, but the sensitive detection of minute changes in spatially dynamic environments to avoid predatory pressure. Dedicated neuronal circuits allowing for such improvement in spatial resolution can be found already on the primary detector level of the brainstem^18^. We therefore propose that the mammalian auditory system evolved for source segregation rather than absolute localization.

## Material and Methods

### Psychophysics

#### Subjects

18 listeners (11 males and 7 females, mean age 24.4 years) participated in the first experiment, eight listeners (five males and three females) participated in the second experiment. All subjects showed normal hearing abilities between 250 Hz and 8000 Hz. All experimental protocols were approved by the ethical board of the Ludwig-Maximilians-Universität medical center and carried out in accordance with the ethical principles of the world medical association for research involving human subjects (Declaration of Helsinki). All subjects gave their written informed consent.

#### Stimuli and experimental design

All stimuli were generated in MatLab (The Mathworks, Inc, Natick, Massachusetts, US) at a sampling rate of 44.1 kHz and were digital-to-analog converted (Audio 2 Dj, Native Instruments GmbH, Berlin, Germany) before being presented over circumaural headphones (HDA 200, Sennheiser Electronic GmbH & Co. KG., Wedemark, Germany) to the listeners. In the first experiment one control and three test conditions were presented. In the control condition (Fig. 2a, top) a single 220-ms target tone (including linear rise-fall times of 10 ms) was presented. In the three test conditions (Fig. 2a, bottom) a 1-s adapter (20 × 50-ms tone pips) preceded the target tone, separated by 500 ms silence. The adapter was presented with one of the following ITDs: (i) −181.4 µs, (ii) 634.9 µs, or (iii) 0 µs. Target tones were presented at 15 different test ITDs ranging from −634.9 μs to 634.9 μs in steps of 90.7 μs.

In the second experiment, the just noticeable difference in ITDs (ITD-jnds) was determined at three lateral (−634.9, +362.8, and +634.9 µs) and one midline ITD, each for a control and a test condition. In the control condition (Fig. 5c, top); two consecutive 160 ms target tones (pause: 140 ms) were presented to the listener. One target tone was presented with a fixed ITD (corresponding to one of the four test ITDs), the other one with an alternating ITD. Alternating ITDs were smaller than the fixed ITD for +362.8,+634.9µs and 0 µs and bigger for the −634.9 µs. In the test condition (Fig. 5c, bottom) a 1-s adapter (20 × 50-ms tone pips) with an ITD of +634.9 µs preceded the target tone pair, separated by 200 ms silence. Both for the control and test condition (i.e. with adapter) ITD-jnds determined at −634.9 µs served as a reference. All stimuli were presented at 200 Hz and 80 dB SPL.

**Figure 6:**
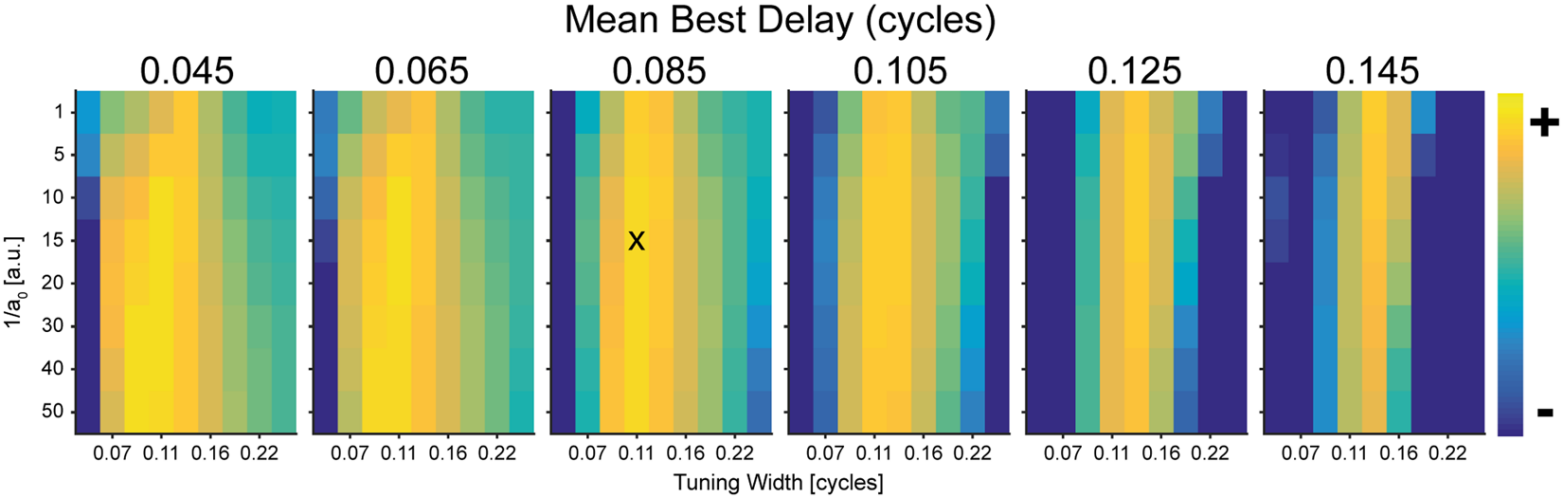
Summed log-likelihoods used for model parameter optimization. The summed log-likelihood (see Methods) for various combinations of tuning width and non-linear weight function (1/a_0_) at six different mean best delays are plotted. Lighter colors denote better overall fitting performance. A broad range of parameter combinations resulted in similarly good model performance (yellow) with insignificant changes in summed log-likelihood values. Settings used throughout the study (black x) were chosen based on highest log-likelihoods given physiologically plausible mean best delays (>0.065 cylces).

#### Procedure

In the first experiment, subjects specified the perceived intracranial position of a target tone by pressing one of 31 linearly arranged buttons (0 being far left and 30 being far right) displayed on a computer screen. One trial consisted of one of the four experimental conditions. Listeners performed three sessions on the three different days. One session always consisted of the control condition followed by one of the three randomly chosen test conditions (150 trials with 10 trials per test ITD). For each condition, test ITDs were presented randomly. To exclude any effect of training listeners did not receive feedback about the presented test ITD and their decision. Moreover, for each listener and session the test condition was chosen randomly. Within one session, the test ITDs were presented randomly.

In the second experiment, listeners specified the perceived direction of a target tone pair (lateralized by ITDs) to determine ITD-jnds. The mean ITD-jnd for each experimental condition was determined by three consecutive runs, using a one-up three-down rule with feedback, as implemented by the MatLab AFC package^49^.

#### Analysis

For each subject and each test ITD, scores values of experiment one were averaged over all trials for the control condition and the test condition within one session (=mean position score). The individual range (mean position score for +634.9 µs minus mean position score for −634.9 µs) was calculated subsequently. To determine the effect of the adapter, a difference score was calculated by subtracting the mean position score of the control condition from the mean position score of the test condition (expressed as the percentage of the subjects’ individual range). Mean position scores, individual ranges and difference scores were calculated for each session (i.e. experimental day) separately. The median difference score for each test condition over all 18 listeners was calculated subsequently.

ITD-jnds for each subject, condition, and tested ITD in experiment two were calculated as a median over nine runs. To determine the effect of a preceding adapter the so-called normalized resolution relative to the control condition was calculated. Therefore, ITD-jnds were normalized to the ITD-jnd determined at −634.9 µs. This was done separately for the control and test condition. Normalized ITD-jnds for the control condition were then subtracted from the test condition.

### Simulation

The fundamental idea of the model is that both brain hemispheres (L: Left, R: Right) independently generate ITD estimates *τ*_L/R_, based on population vector decoding of the activities in the binaural nuclei (MSO, lLSO). The two estimates are then combined to yield a hemispherically balanced ITD estimate

1 $$\alpha =\text{arg}[g({a}_{{\rm{R}}})\,\exp (i{\tau }_{{\rm{R}}})+g({a}_{{\rm{L}}})\,\exp (i{\tau }_{{\rm{L}}})]$$

in which *a*_h_ are the total spike counts of the SOC afferents to midbrain hemisphere h (Fig. 1c) and *g(a)* = exp(*a/a*_0_) a monotonically increasing function (Numerical simulations are performed for *a*_0_ = 0.07).

#### Intra-Hemispheric Estimates

In analogy to the hemispheric-balance estimate *α*, also the intra-hemispheric estimates *ϕ*_*h*_ are assumed to arise from a population vector decoder. An essential parameter for such a decoder is the number of populations (channels) that contribute to it. While, in principle, each neuron in the respective nuclei could act as an individual channel (following the labeled-line code proposed by Jeffress^6^), other studies argued that fewer channels (one or two per hemisphere) might actually be sufficient^11,12,50–53^. Since the behavioural and functional differences between a low number of channels per hemisphere and a full-blown multi-channel code in which all neurons vote for their individual ITD^54,55^ are still unclear, we rather follow an argument that is based on anatomy. Particularly, we center our considerations on the feedback systems of the MSO and low-frequency LSO (lLSO) that act on their presynaptic GABA-B receptors and mediate adaptation^18,20^. At the MSO, GABA is released into the extracellular medium by varicosities arising from the GABA-ergic SPN neurons^18^. This GABA release is not cell specific but rather affects a whole sample of the MSO neurons probably a large fraction of them within a best frequency band. For the lLSO the GABA feedback is more cell-specific since there GABA is released from the LSO dendrites^20^. However, also there GABA is released extra-synaptically and will also bind to GABA-B receptors on synapses of neighboring neurons. Both feedback mechanisms thus treat a subset of neurons in MSO and lLSO as computational entity and, thus, we assume that there are only two channels per hemisphere, one associated to MSO and the other one to lLSO. Note, that MSO and LSO have opposite hemispherical preferences, yet their projections to the IC lead to similar hemispherical preferences there because MSO projects ipsilaterally and LSO contralaterally.

If we denote the respective neuronal activities by μ_h_ (MSO) and λ_h_ (lLSO) the hemispheric estimate can thus be computed as

2 $${\varphi }_{{\rm{h}}}=\text{arg}[{\mu }_{{\rm{h}}}\exp (\pm {\rm{i}}{\varphi }_{{\rm{\mu }}})+{\lambda }_{{\rm{h}}}\exp (\pm {\rm{i}}{\varphi }_{{\rm{\lambda }}})]$$

where the argument of the exponential function is positive for h = R and negative for h = L. The parameters *ϕ*_λ_ > *ϕ*_μ_ > 0 denote the azimuthal angles the population of MSO and lLSO neurons vote for. We made a straight-forward symmetric choice *ϕ*_μ_=π/4 *ϕ*_λ_=π/2. The estimates *ϕ*_*h*_ in equation (2) do not represent the azimuthal angle or ITD directly, they are conceived as a one-dimensional isomorphism of ITD that may be learned by the readout structures. More specifically, for each ITD (in steps of 5µs) in a control situation (without adapter) we compute *ϕ*_*h*_ corresponding to this ITD and subsequently use these (*ϕ*_*h*_, ITD) pairs as a lookup table to estimate the ITD τ_h_ from the corresponding *ϕ*_*h*_ value in the adapted case. Consequently, the specific choice of *ϕ*_μ_ and *ϕ*_λ_ does not affect the results (as long as the two vectors exp(i*ϕ*_μ_) and exp(i*ϕ*_λ_) are linearly independent). The activities variables μ_h_ and λ_h_ might not necessarily be the spike counts but some nonlinear function of it. In this paper we introduce a threshold and saturation by choosing μ_h_ = tanh(k_h_ − 0.05) for k_h_ > 0.05 and μ_h_ = 0 otherwise, where k_h_ denotes the total a scaled spike count of the MSO neurons in one frequency band and the λ_h_ is computed analogously for the spike count of the lLSO population.

It is important to note that the present version of the model does not include level dependence. It therefore assumes that the outcome is level-invariant. The latter can be achieved for the hemispheric estimates *ϕ*_*h*_ if one assumes that the rates *μ*_h_ and *λ*_h_ in both hemispheres change by the same factor upon a change in level. Moreover, the invariance property also requires that for the non-linear weighting function g, *a*_0_ is increased by the same factor as the firing rates *a*.

#### Two further decoding models

the hemispheric difference model and the labeled line model are also used in this study for comparison. They both rely on variants of equation (2). For the hemispheric difference model the rates *μ*_h_ and *λ*_h_ are replaced by the populations rates *μ*_R_ and *μ*_L_ of the two hemispheres (MSO and lLSO summed together) and the respective directions *ϕ*_λ_ and *ϕ*_μ_ are taken as ±45° resulting in *α* = arctan[(*μ*_R_ − *μ*_L_)/(*μ*_R_ + *μ*_L_)], which around midline matches the normalized difference of the firing rates two hemispheres. The angle *α* is then transformed to an ITD according to the same learning procedure as in the hemispheric balance model.

The labeled line model extends the sum in eq. (2) to as many summands as there are neurons (2000 per hemisphere; 1000 MSO and 1000 lLSO), with respective directions *ϕ*_*i*_ that corresponds to the individual neurons best delay.

#### Modelling Firing Rates

The model for the firing rates of MSO and lLSO neurons underlying the population code takes into account the best frequency of the neuron as well as the frequency-dependence of best ITDs^4,5^

3 $${\rm{best}}\,{\rm{ITD}}\,(f)={\rm{CD}}+\mathrm{CP}/f{,}$$

in which CD and CP denote the characteristic delay and characteristic phase, respectively. The populations of MSO and lLSO neurons differ in the distributions of CPs and CDs. For MSO neurons we assume the CPs to be uniformly distributed between −1/4 and +1/4 of a cycle and the best phases (best ITDs at best frequency times best frequency) are normally distributed with mean 0.085 and standard deviation 0.05 cycles^5^. The CDs follow from eq. (3). For the lLSO population we take CPs uniformly distributed between +1/4 and +3/4 of a cycle and the best phases normally distributed with mean 0.775 cycles and standard deviation 0.05 cycles (cf.^32^). Note that 0.775 would correspond to a best phase of 0.225 on the level of the contralateral midbrain. The parameter choice implements the negative correlation between CP and CD observed in the DNLL of gerbils^52,56^ see Supplementary Fig. 1a.

The rate *r*(*f*, ITD) as a function of stimulus frequency f and ITD is thus modelled as

4 $$r(f,{\rm{ITD}})=A(f)\exp \{[\cos [2{\rm{\pi }}{f}({\rm{ITD}}-{\rm{bestITD}}(f))]-1]/{(2{\rm{\pi }}{\sigma })}^{2}\}$$

where *A*(*f*) = (*f*/BF)^sign(BF-*f*)*κ*^ introduces the best frequency BF of the neuron as the frequency for which the rate amplitude *A* is maximal and is applied to all simulated neurons.

The two remaining free parameters σ and κ determine the width of the ITD tuning curve and the spectral width of the receptive field, respectively. All numerical results in this paper are obtained for *σ* = 0.111 and *κ* = 1/ln(1.3) unless mentioned otherwise; see Supplementary Fig. 1b.

#### Modelling Adaptation

Motivated by the anatomy of the feedback loops in the SOC (see Methods), we assume adaptation to act on the level of populations, i.e., the feedback signal *d* is the same for every neuron in the same frequency band of the respective MSO or lLSO. Since GABA-B acts on the presynaptic side, we assume the adaptation to multiplicatively scale the neuronal activity and, thus, for every neuron *i* we obtain as the firing rate as

5 $$Ri(f,{\rm{ITD}})={r}_{i}(f,{\rm{ITD}}){d}_{i}({\boldsymbol{R}})$$

where *r* denotes the non-adapted rate from eq. (4) and ***R*** is the vector of all firing rate in the ipsilateral MSO or lLSO, respectively. Owing to its recursive nature, we solve eq. (5) implicitly using Newton’s method. The adaptation *d*_i_ of the i-th frequency-band (Supplementary Fig. 1c_1_) is thereby modelled as

6 $${d}_{i}({\boldsymbol{R}})=0.5\,\tanh [-\beta ({(W{\boldsymbol{R}})}_{{\rm{i}}}-\theta )]+0.5,$$

with *β* = 1/2, and a feedback matrix W that incorporates frequency-specificity of the feedback adaptation by

7 $${W}_{ij}={({f}_{i}/{f}_{j})}^{sign(fj-fi)/(4\mathrm{ln}1.3)}.$$

The best frequencies of the neurons i and j are thereby denoted by *f*_i_ and *f*_j_, respectively. The threshold *θ* equals 5 for the MSO population and 4 for the lLSO population to account for the lower average firing rate in the lLSO populations (Supplementary Fig. 1c_2_).

#### Analysis of Model Perfomance

To evaluate the goodness of the mdeolodel fits to the behavioral data of we calculated the Pearson’s correlation coefficient (ρ with corresponding p-values for each model and each adapter condition, respectively (see Fig. Figure 4). Positive values of ρ denote a positive linear correlation, 0 denote no correlation and negative values give a negative correlation between data and model fit. We further determined the log-likelihood values of how well the model predicts the data.To this end we used the mean positional estimate m(ITD) and variance v(ITD) from the psychoacoustic data and defined likelihoods for the model predictions *α* as

8 $${\rm{L}}(\alpha )={{\rm{\Pi }}}_{{\rm{ITD}}}\exp [-{(\alpha -m({\rm{ITD}}))}^{2}/(2v(ITD))]{(2{\rm{\pi }}v({\rm{ITD}}))}^{-1/2}$$

logarithmic probability density function)Log_10_ likelihoods were computed for at each of the 15 test-ITDs separately and subsequently summed these values for each model andeach adapter condition and subsequently summed to obtain one likelihood value for each model and parameter choice (See Supplementary Table 1). Here, higher log likelihood values values denote better model fits.

We determined the log-likelihood value (using the logarithmic probability density function provided by Matlab, The Mathworks) at each of the 15 test-ITDs separately. In short, this procedure determines the likelihood of an observed value (in our case the model prediction at a given ITD) to stem from a distribution with defined mean and standard deviation (the observed behavioral data). Here, higher values denote better model fits. Log-likelihood were first summed across all test-ITD. subsequently summed these values for each model and adapter condition (See Supplementary Table 1). Here, higher values denote better model fits.To determine an unbiased optimal combination of mean best delay (6 different settings), tuning width (9 different settings) and the non-linear weighting function (8 different settings), these log-likelihood values were summed over all adapter condition and model version for each parameter combination separately. This analysis revealed a broad range of parameter combinations that resulted in similarly good model performance with insignificant changes in summed log-likelihood values (Fig. 6). Settings used throughout the study were chosen based on highest log-likelihoods given physiologically plausible mean best delays.

## Electronic supplementary material

Supplementary information
