# Supplementary material for: A novel concept for dynamic adjustment of auditory space
Source: Sci Rep. 2018 May 29;8:8335. doi: 10.1038/s41598-018-26690-0 (PMC5974081; doi:10.1038/s41598-018-26690-0)
Supplement: Supplementary file 1 — Supplementary information [file 41598_2018_26690_MOESM1_ESM.docx]

**Supplementary information**

**A novel concept for dynamic adjustment of auditory space**

Lingner, A^1+^, Pecka M^1+^, Leibold C^2^, Grothe B^1*^

^1^ Division of Neurobiology, Department Biology II

Ludwig-Maximilians-Universitaet Muenchen

Großhaderner Str. 2-4, D-82152 Martinsried-Planegg, Germany

^2^ Bernstein Center for Computational Neuroscience Munich,

Großhaderner Straße 2-4, D-82152 Martinsried, Germany

^+^ contributed equally

*Corresponding author: grothe@lmu.de

**FIGURES Supplement**


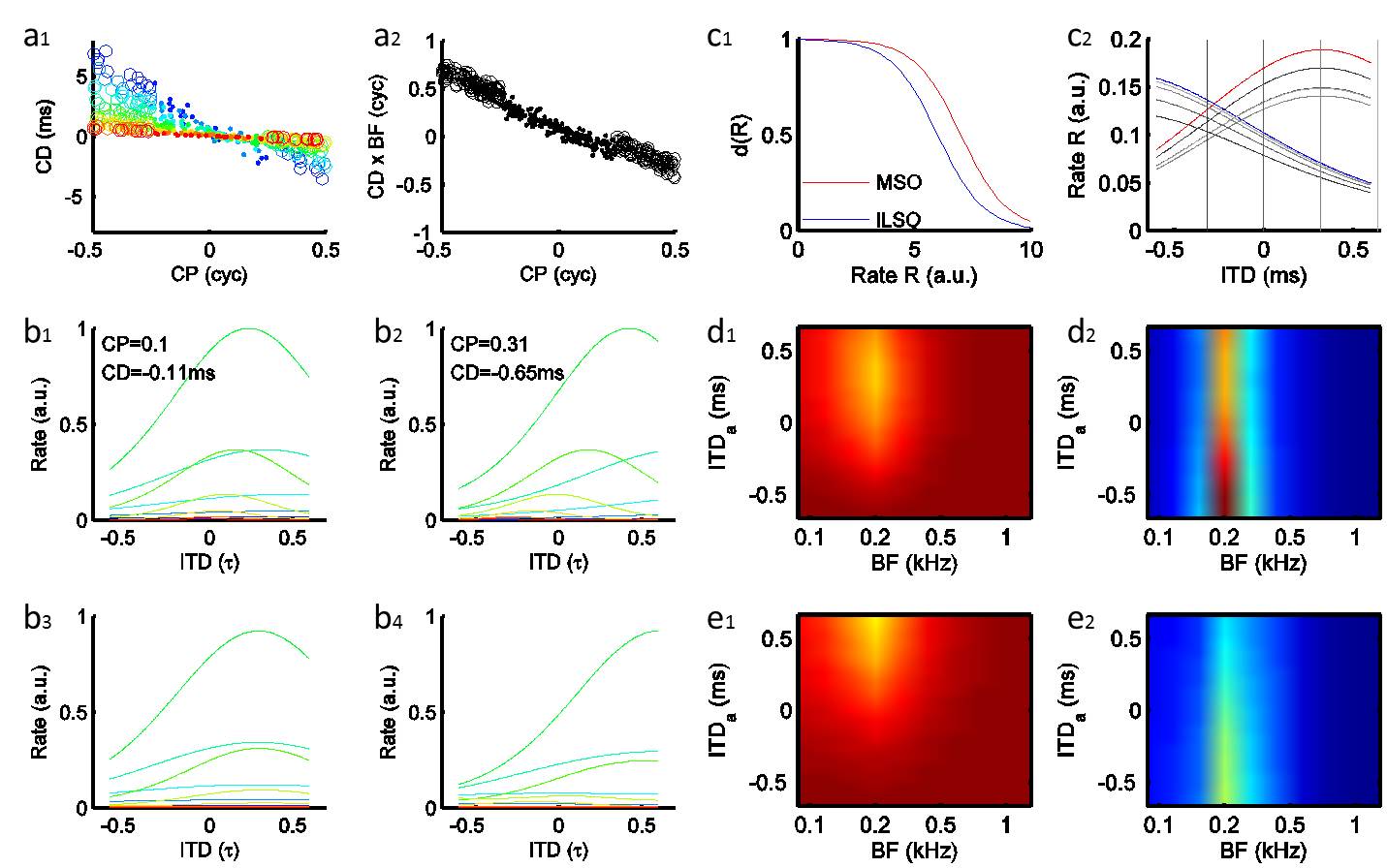
 Supplementary Figure 1: COMPUTATIONAL MODEL. (**a**) Distribution of characteristic delay (CD), phase (CP) and best frequency (10 frequency channels from blue: 100Hz to red: 1kHz) in the simulated population of neurons. Neurons with CP between -1/4 and +1/4 cycles are identified as peakers (dots, 200 per frequency channel) and are assumed to reside in the MSO, the others as troughers (circles, 200 per frequency channel) and assumed to reside in the lLSO. (**b**_1,2_) Tone delay functions for an example peaker and trougher neuron. (**b**_3,4_) Tone delay functions for the whole populations of peakers and troughers, respectively. The colors indicate the stimulus frequency (same convention as in **a**). (**c**_1_) Adaptive attenuation *d* as a function of firing rate. (**c**_2_) Firing rate of right MSO and left lLSO population for different adapter positions (grey level of vertical lines corresponds to grey levels of firing rate). Blue and red curves depict unadapted response. (**d**) Adapter attenuation (**d**_1_) and firing rate (**d**_2_) of the all right-hemisphere peakers (MSO) averaged over frequency band for a 200 Hz adapter at ITD_a_ and a 200Hz test tone at ITD = -575 μs. Positive adapter ITD_a_s yield stronger attenuations. (**e**) Same as **d** for right hemisphere troughers (lLSO).
